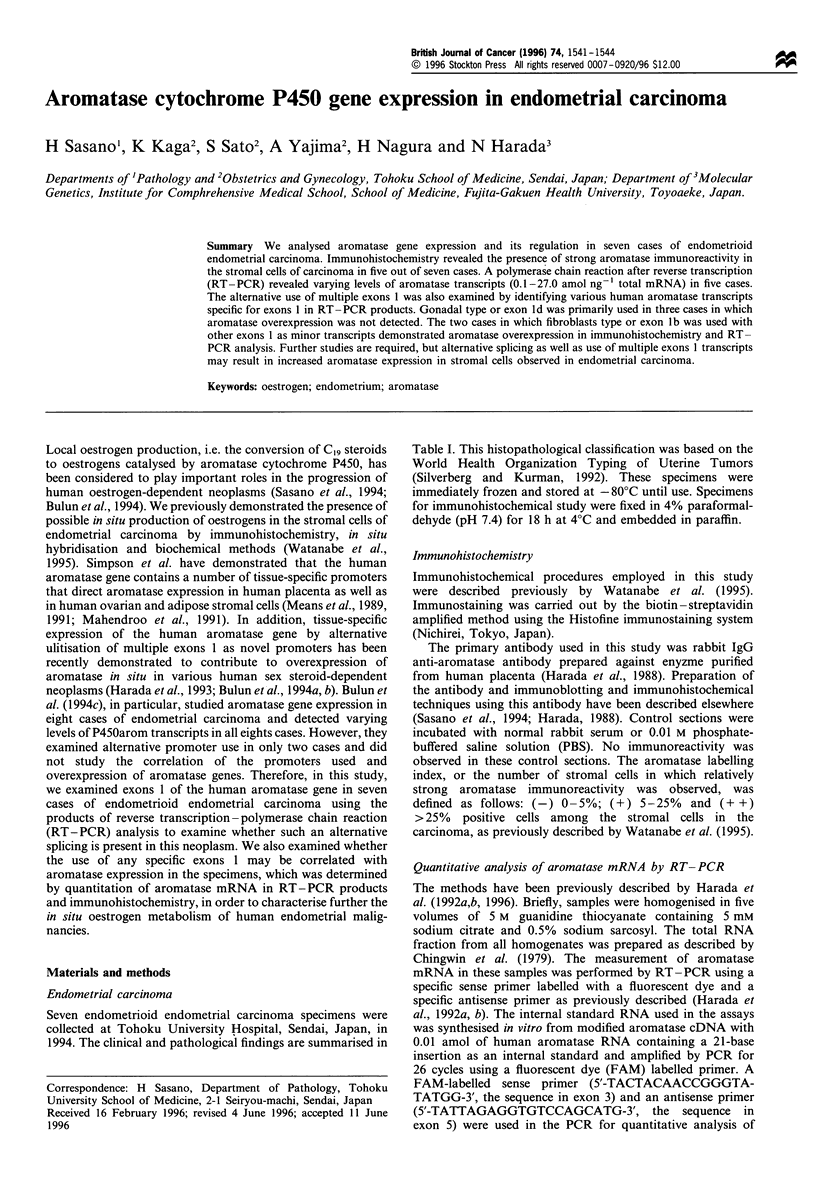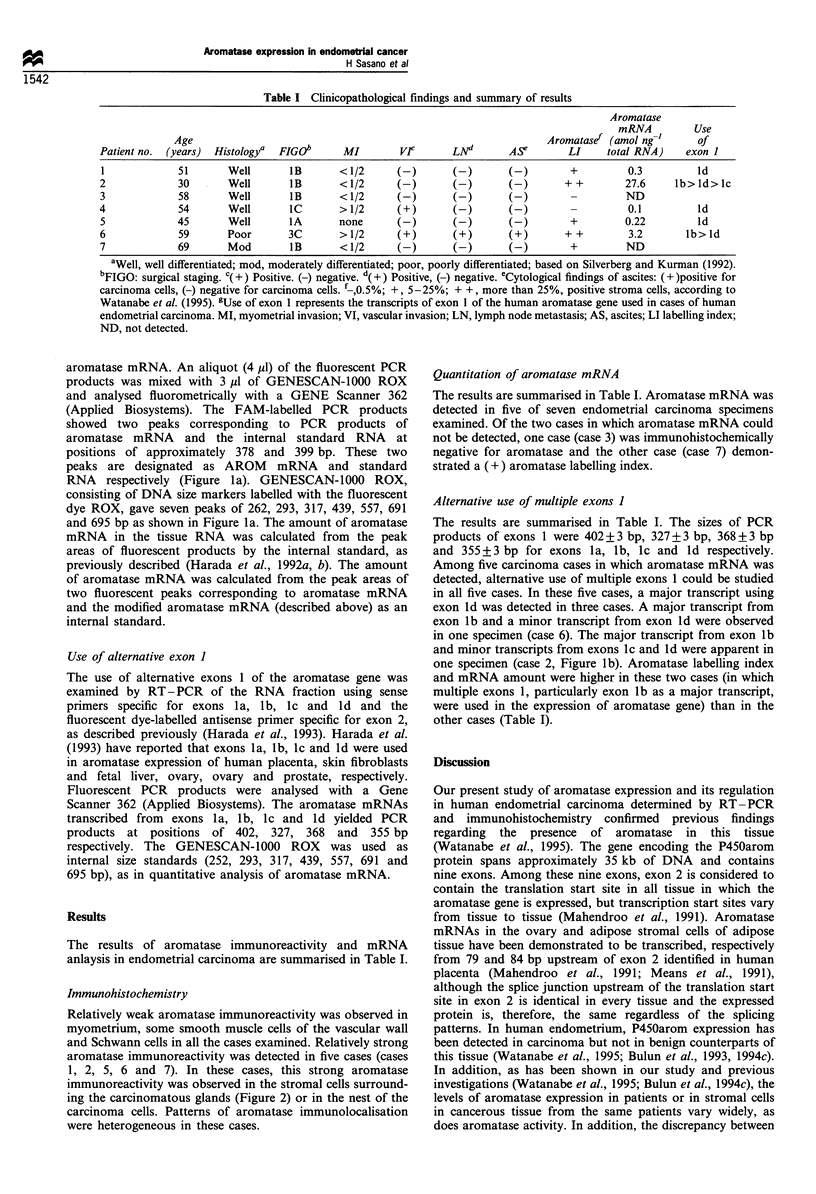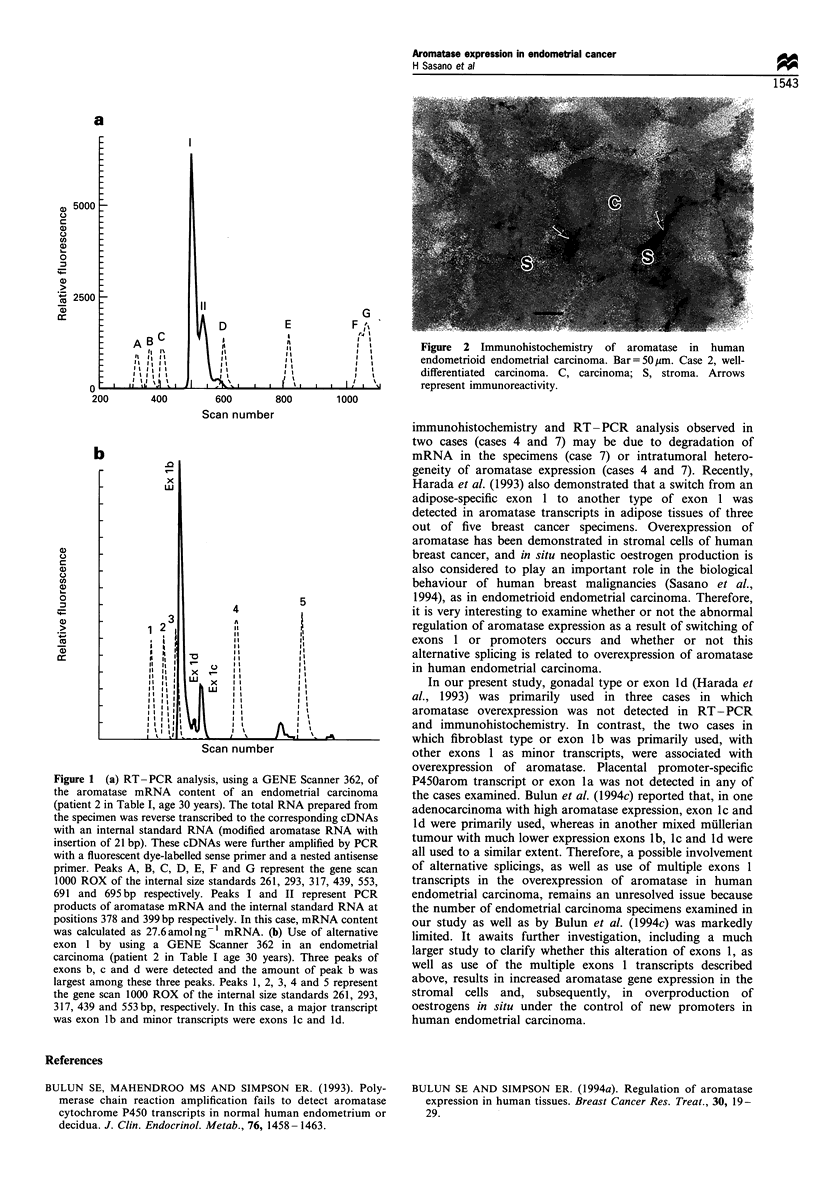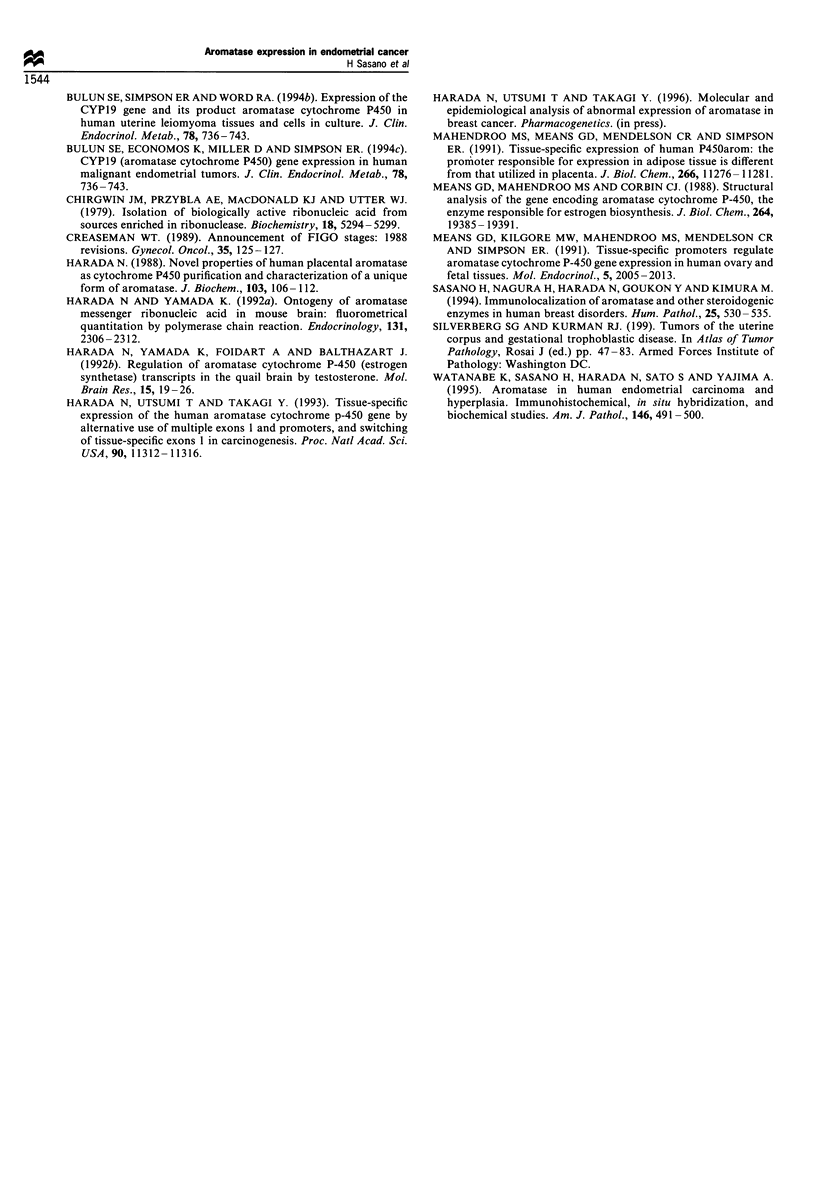# Aromatase cytochrome P450 gene expression in endometrial carcinoma.

**DOI:** 10.1038/bjc.1996.586

**Published:** 1996-11

**Authors:** H. Sasano, K. Kaga, S. Sato, A. Yajima, H. Nagura, N. Harada

**Affiliations:** Department of Pathology, Tohoku School of Medicine, Sendai, Japan.

## Abstract

**Images:**


British Journal of Cancer (1996) 74, 1541-1544
© 1996 Stockton Press All rights reserved 0007-0920/96 $12.00

### Aromatase cytochrome P450 gene expression in endometrial carcinoma

H Sasano[1], K Kaga[2], S Sato[2], A Yajima[2], H Nagura and N Harada[3]

*Departments of [1]Pathology and [2]Obstetrics and Gynecology, Tohoku School of Medicine, Sendai, Japan; Department of [3]Molecular Genetics, Institute for Comphrehensive Medical School, School of Medicine, Fujita-Gakuen Health University, Toyoaeke, Japan.*

**Summary** We analysed aromatase gene expression and its regulation in seven cases of endometrioid endometrial carcinoma. Immunohistochemistry revealed the presence of strong aromatase immunoreactivity in the stromal cells of carcinoma in five out of seven cases. A polymerase chain reaction after reverse transcription (RT-PCR) revealed varying levels of aromatase transcripts (0.1-27.0 amol $ng^{-1}$ total mRNA) in five cases. The alternative use of multiple exons 1 was also examined by identifying various human aromatase transcripts specific for exons 1 in RT-PCR products. Gonadal type or exon 1d was primarily used in three cases in which aromatase overexpression was not detected. The two cases in which fibroblasts type or exon 1b was used with other exons 1 as minor transcripts demonstrated aromatase overexpression in immunohistochemistry and RT-PCR analysis. Further studies are required, but alternative splicing as well as use of multiple exons 1 transcripts may result in increased aromatase expression in stromal cells observed in endometrial carcinoma.

**Keywords:** oestrogen; endometrium; aromatase

Local oestrogen production, i.e. the conversion of $C_{19}$ steroids to oestrogens catalysed by aromatase cytochrome P450, has been considered to play important roles in the progression of human oestrogen-dependent neoplasms (Sasano *et al.*, 1994; Bulun *et al.*, 1994). We previously demonstrated the presence of possible *in situ* production of oestrogens in the stromal cells of endometrial carcinoma by immunohistochemistry, *in situ* hybridisation and biochemical methods (Watanabe *et al.*, 1995). Simpson *et al.* have demonstrated that the human aromatase gene contains a number of tissue-specific promoters that direct aromatase expression in human placenta as well as in human ovarian and adipose stromal cells (Means *et al.*, 1989, 1991; Mahendroo *et al.*, 1991). In addition, tissue-specific expression of the human aromatase gene by alternative ulitisation of multiple exons 1 as novel promoters has been recently demonstrated to contribute to overexpression of aromatase *in situ* in various human sex steroid-dependent neoplasms (Harada *et al.*, 1993; Bulun *et al.*, 1994*a*, *b*). Bulun *et al.* (1994*c*), in particular, studied aromatase gene expression in eight cases of endometrial carcinoma and detected varying levels of P450arom transcripts in all eights cases. However, they examined alternative promoter use in only two cases and did not study the correlation of the promoters used and overexpression of aromatase genes. Therefore, in this study, we examined exons 1 of the human aromatase gene in seven cases of endometrioid endometrial carcinoma using the products of reverse transcription-polymerase chain reaction (RT-PCR) analysis to examine whether such an alternative splicing is present in this neoplasm. We also examined whether the use of any specific exons 1 may be correlated with aromatase expression in the specimens, which was determined by quantitation of aromatase mRNA in RT-PCR products and immunohistochemistry, in order to characterise further the *in situ* oestrogen metabolism of human endometrial malignancies.

### Materials and methods

### *Endometrial carcinoma*

Seven endometrioid endometrial carcinoma specimens were collected at Tohoku University Hospital, Sendai, Japan, in 1994. The clinical and pathological findings are summarised in Table I. This histopathological classification was based on the World Health Organization Typing of Uterine Tumors (Silverberg and Kurman, 1992). These specimens were immediately frozen and stored at -80°C until use. Specimens for immunohistochemical study were fixed in 4% paraformaldehyde (pH 7.4) for 18 h at 4°C and embedded in paraffin.

### *Immunohistochemistry*

Immunohistochemical procedures employed in this study were described previously by Watanabe *et al.* (1995). Immunostaining was carried out by the biotin-streptavidin amplified method using the Histofine immunostaining system (Nichirei, Tokyo, Japan).

The primary antibody used in this study was rabbit IgG anti-aromatase antibody prepared against enyzme purified from human placenta (Harada *et al.*, 1988). Preparation of the antibody and immunoblotting and immunohistochemical techniques using this antibody have been described elsewhere (Sasano *et al.*, 1994; Harada, 1988). Control sections were incubated with normal rabbit serum or 0.01 M phosphate-buffered saline solution (PBS). No immunoreactivity was observed in these control sections. The aromatase labelling index, or the number of stromal cells in which relatively strong aromatase immunoreactivity was observed, was defined as follows: (-) 0-5%; (+) 5-25% and (++) >25% positive cells among the stromal cells in the carcinoma, as previously described by Watanabe *et al.* (1995).

### *Quantitative analysis of aromatase mRNA by RT-PCR*

The methods have been previously described by Harada *et al.* (1992*a*,*b*, 1996). Briefly, samples were homogenised in five volumes of 5 M guanidine thiocyanate containing 5 mM sodium citrate and 0.5% sodium sarcosyl. The total RNA fraction from all homogenates was prepared as described by Chingwin *et al.* (1979). The measurement of aromatase mRNA in these samples was performed by RT-PCR using a specific sense primer labelled with a fluorescent dye and a specific antisense primer as previously described (Harada *et al.*, 1992*a*, *b*). The internal standard RNA used in the assays was synthesised *in vitro* from modified aromatase cDNA with 0.01 amol of human aromatase RNA containing a 21-base insertion as an internal standard and amplified by PCR for 26 cycles using a fluorescent dye (FAM) labelled primer. A FAM-labelled sense primer (5'-TACTACAACCGGGTA-TATGG-3', the sequence in exon 3) and an antisense primer (5'-TATTAGAGGTGTCCAGCATG-3', the sequence in exon 5) were used in the PCR for quantitative analysis of

Correspondence: H Sasano, Department of Pathology, Tohoku University School of Medicine, 2-1 Seiryou-machi, Sendai, Japan
Received 16 February 1996; revised 4 June 1996; accepted 11 June 1996

**Table I** Clinicopathological findings and summary of results

| Patient no. | Age (years) | Histology[a] | FIGO[b] | MI | VI[c] | LN[d] | AS[e] | Aromatase[f] LI | Aromatase mRNA (amol ng$^{-1}$ total RNA) | Use of exon 1 |
|---|---|---|---|---|---|---|---|---|---|---|
| 1 | 51 | Well | 1B | <1/2 | (−) | (−) | (−) | + | 0.3 | 1d |
| 2 | 30 | Well | 1B | <1/2 | (−) | (−) | (−) | + + | 27.6 | 1b>1d>1c |
| 3 | 58 | Well | 1B | <1/2 | (−) | (−) | (−) | − | ND | |
| 4 | 54 | Well | 1C | >1/2 | (+) | (−) | (−) | − | 0.1 | 1d |
| 5 | 45 | Well | 1A | none | (−) | (−) | (−) | + | 0.22 | 1d |
| 6 | 59 | Poor | 3C | >1/2 | (+) | (+) | (+) | + + | 3.2 | 1b>1d |
| 7 | 69 | Mod | 1B | <1/2 | (−) | (−) | (−) | + | ND | |

[a]Well, well differentiated; mod, moderately differentiated; poor, poorly differentiated; based on Silverberg and Kurman (1992). [b]FIGO: surgical staging. [c](+) Positive. (–) negative. [d](+) Positive, (–) negative. [e]Cytological findings of ascites: (+)positive for carcinoma cells, (–) negative for carcinoma cells. [f]–,0.5%; +, 5–25%; + +, more than 25%, positive stroma cells, according to Watanabe *et al.* (1995). [g]Use of exon 1 represents the transcripts of exon 1 of the human aromatase gene used in cases of human endometrial carcinoma. MI, myometrial invasion; VI, vascular invasion; LN, lymph node metastasis; AS, ascites; LI labelling index; ND, not detected.

aromatase mRNA. An aliquot (4 μl) of the fluorescent PCR products was mixed with 3 μl of GENESCAN-1000 ROX and analysed fluorometrically with a GENE Scanner 362 (Applied Biosystems). The FAM-labelled PCR products showed two peaks corresponding to PCR products of aromatase mRNA and the internal standard RNA at positions of approximately 378 and 399 bp. These two peaks are designated as AROM mRNA and standard RNA respectively (Figure 1a). GENESCAN-1000 ROX, consisting of DNA size markers labelled with the fluorescent dye ROX, gave seven peaks of 262, 293, 317, 439, 557, 691 and 695 bp as shown in Figure 1a. The amount of aromatase mRNA in the tissue RNA was calculated from the peak areas of fluorescent products by the internal standard, as previously described (Harada *et al.*, 1992*a*, *b*). The amount of aromatase mRNA was calculated from the peak areas of two fluorescent peaks corresponding to aromatase mRNA and the modified aromatase mRNA (described above) as an internal standard.

### *Use of alternative exon 1*

The use of alternative exons 1 of the aromatase gene was examined by RT–PCR of the RNA fraction using sense primers specific for exons 1a, 1b, 1c and 1d and the fluorescent dye-labelled antisense primer specific for exon 2, as described previously (Harada *et al.*, 1993). Harada *et al.* (1993) have reported that exons 1a, 1b, 1c and 1d were used in aromatase expression of human placenta, skin fibroblasts and fetal liver, ovary, ovary and prostate, respectively. Fluorescent PCR products were analysed with a Gene Scanner 362 (Applied Biosystems). The aromatase mRNAs transcribed from exons 1a, 1b, 1c and 1d yielded PCR products at positions of 402, 327, 368 and 355 bp respectively. The GENESCAN-1000 ROX was used as internal size standards (252, 293, 317, 439, 557, 691 and 695 bp), as in quantitative analysis of aromatase mRNA.

### Results

The results of aromatase immunoreactivity and mRNA anlaysis in endometrial carcinoma are summarised in Table I.

### *Immunohistochemistry*

Relatively weak aromatase immunoreactivity was observed in myometrium, some smooth muscle cells of the vascular wall and Schwann cells in all the cases examined. Relatively strong aromatase immunoreactivity was detected in five cases (cases 1, 2, 5, 6 and 7). In these cases, this strong aromatase immunoreactivity was observed in the stromal cells surrounding the carcinomatous glands (Figure 2) or in the nest of the carcinoma cells. Patterns of aromatase immunolocalisation were heterogeneous in these cases.

### *Quantitation of aromatase mRNA*

The results are summarised in Table I. Aromatase mRNA was detected in five of seven endometrial carcinoma specimens examined. Of the two cases in which aromatase mRNA could not be detected, one case (case 3) was immunohistochemically negative for aromatase and the other case (case 7) demonstrated a (+) aromatase labelling index.

### *Alternative use of multiple exons 1*

The results are summarised in Table I. The sizes of PCR products of exons 1 were 402±3 bp, 327±3 bp, 368±3 bp and 355±3 bp for exons 1a, 1b, 1c and 1d respectively. Among five carcinoma cases in which aromatase mRNA was detected, alternative use of multiple exons 1 could be studied in all five cases. In these five cases, a major transcript using exon 1d was detected in three cases. A major transcript from exon 1b and a minor transcript from exon 1d were observed in one specimen (case 6). The major transcript from exon 1b and minor transcripts from exons 1c and 1d were apparent in one specimen (case 2, Figure 1b). Aromatase labelling index and mRNA amount were higher in these two cases (in which multiple exons 1, particularly exon 1b as a major transcript, were used in the expression of aromatase gene) than in the other cases (Table I).

### Discussion

Our present study of aromatase expression and its regulation in human endometrial carcinoma determined by RT–PCR and immunohistochemistry confirmed previous findings regarding the presence of aromatase in this tissue (Watanabe *et al.*, 1995). The gene encoding the P450arom protein spans approximately 35 kb of DNA and contains nine exons. Among these nine exons, exon 2 is considered to contain the translation start site in all tissue in which the aromatase gene is expressed, but transcription start sites vary from tissue to tissue (Mahendroo *et al.*, 1991). Aromatase mRNAs in the ovary and adipose stromal cells of adipose tissue have been demonstrated to be transcribed, respectively from 79 and 84 bp upstream of exon 2 identified in human placenta (Mahendroo *et al.*, 1991; Means *et al.*, 1991), although the splice junction upstream of the translation start site in exon 2 is identical in every tissue and the expressed protein is, therefore, the same regardless of the splicing patterns. In human endometrium, P450arom expression has been detected in carcinoma but not in benign counterparts of this tissue (Watanabe *et al.*, 1995; Bulun *et al.*, 1993, 1994*c*). In addition, as has been shown in our study and previous investigations (Watanabe *et al.*, 1995; Bulun *et al.*, 1994*c*), the levels of aromatase expression in patients or in stromal cells in cancerous tissue from the same patients vary widely, as does aromatase activity. In addition, the discrepancy between

**Figure 2** Immunohistochemistry of aromatase in human endometrioid endometrial carcinoma. Bar = 50 $\mu$m. Case 2, well-differentiated carcinoma. C, carcinoma; S, stroma. Arrows represent immunoreactivity.

immunohistochemistry and RT–PCR analysis observed in two cases (cases 4 and 7) may be due to degradation of mRNA in the specimens (case 7) or intratumoral heterogeneity of aromatase expression (cases 4 and 7). Recently, Harada *et al.* (1993) also demonstrated that a switch from an adipose-specific exon 1 to another type of exon 1 was detected in aromatase transcripts in adipose tissues of three out of five breast cancer specimens. Overexpression of aromatase has been demonstrated in stromal cells of human breast cancer, and *in situ* neoplastic oestrogen production is also considered to play an important role in the biological behaviour of human breast malignancies (Sasano *et al.*, 1994), as in endometrioid endometrial carcinoma. Therefore, it is very interesting to examine whether or not the abnormal regulation of aromatase expression as a result of switching of exons 1 or promoters occurs and whether or not this alternative splicing is related to overexpression of aromatase in human endometrial carcinoma.

In our present study, gonadal type or exon 1d (Harada *et al.*, 1993) was primarily used in three cases in which aromatase overexpression was not detected in RT–PCR and immunohistochemistry. In contrast, the two cases in which fibroblast type or exon 1b was primarily used, with other exons 1 as minor transcripts, were associated with overexpression of aromatase. Placental promoter-specific P450arom transcript or exon 1a was not detected in any of the cases examined. Bulun *et al.* (1994*c*) reported that, in one adenocarcinoma with high aromatase expression, exon 1c and 1d were primarily used, whereas in another mixed müllerian tumour with much lower expression exons 1b, 1c and 1d were all used to a similar extent. Therefore, a possible involvement of alternative splicings, as well as use of multiple exons 1 transcripts in the overexpression of aromatase in human endometrial carcinoma, remains an unresolved issue because the number of endometrial carcinoma specimens examined in our study as well as by Bulun *et al.* (1994*c*) was markedly limited. It awaits further investigation, including a much larger study to clarify whether this alteration of exons 1, as well as use of the multiple exons 1 transcripts described above, results in increased aromatase gene expression in the stromal cells and, subsequently, in overproduction of oestrogens *in situ* under the control of new promoters in human endometrial carcinoma.

### References

BULUN SE, MAHENDROO MS AND SIMPSON ER. (1993). Polymerase chain reaction amplification fails to detect aromatase cytochrome P450 transcripts in normal human endometrium or decidua. *J. Clin. Endocrinol. Metab.*, **76**, 1458–1463.

BULUN SE AND SIMPSON ER. (1994*a*). Regulation of aromatase expression in human tissues. *Breast Cancer Res. Treat.*, **30**, 19–29.

BULUN SE, SIMPSON ER AND WORD RA. (1994*b*). Expression of the CYP19 gene and its product aromatase cytochrome P450 in human uterine leiomyoma tissues and cells in culture. *J. Clin. Endocrinol. Metab.*, **78,** 736–743.

BULUN SE, ECONOMOS K, MILLER D AND SIMPSON ER. (1994*c*). CYP19 (aromatase cytochrome P450) gene expression in human malignant endometrial tumors. *J. Clin. Endocrinol. Metab.*, **78,** 736–743.

CHIRGWIN JM, PRZYBLA AE, MACDONALD KJ AND UTTER WJ. (1979). Isolation of biologically active ribonucleic acid from sources enriched in ribonuclease. *Biochemistry*, **18,** 5294–5299.

CREASEMAN WT. (1989). Announcement of FIGO stages: 1988 revisions. *Gynecol. Oncol.*, **35,** 125–127.

HARADA N. (1988). Novel properties of human placental aromatase as cytochrome P450 purification and characterization of a unique form of aromatase. *J. Biochem.*, **103,** 106–112.

HARADA N AND YAMADA K. (1992*a*). Ontogeny of aromatase messenger ribonucleic acid in mouse brain: fluorometrical quantitation by polymerase chain reaction. *Endocrinology*, **131,** 2306–2312.

HARADA N, YAMADA K, FOIDART A AND BALTHAZART J. (1992*b*). Regulation of aromatase cytochrome P-450 (estrogen synthetase) transcripts in the quail brain by testosterone. *Mol. Brain Res.*, **15,** 19–26.

HARADA N, UTSUMI T AND TAKAGI Y. (1993). Tissue-specific expression of the human aromatase cytochrome p-450 gene by alternative use of multiple exons 1 and promoters, and switching of tissue-specific exons 1 in carcinogenesis. *Proc. Natl Acad. Sci. USA*, **90,** 11312–11316.

HARADA N, UTSUMI T AND TAKAGI Y. (1996). Molecular and epidemiological analysis of abnormal expression of aromatase in breast cancer. *Pharmacogenetics*. (in press).

MAHENDROO MS, MEANS GD, MENDELSON CR AND SIMPSON ER. (1991). Tissue-specific expression of human P450arom: the promoter responsible for expression in adipose tissue is different from that utilized in placenta. *J. Biol. Chem.*, **266,** 11276–11281.

MEANS GD, MAHENDROO MS AND CORBIN CJ. (1988). Structural analysis of the gene encoding aromatase cytochrome P-450, the enzyme responsible for estrogen biosynthesis. *J. Biol. Chem.*, **264,** 19385–19391.

MEANS GD, KILGORE MW, MAHENDROO MS, MENDELSON CR AND SIMPSON ER. (1991). Tissue-specific promoters regulate aromatase cytochrome P-450 gene expression in human ovary and fetal tissues. *Mol. Endocrinol.*, **5,** 2005–2013.

SASANO H, NAGURA H, HARADA N, GOUKON Y AND KIMURA M. (1994). Immunolocalization of aromatase and other steroidogenic enzymes in human breast disorders. *Hum. Pathol.*, **25,** 530–535.

SILVERBERG SG AND KURMAN RJ. (199). Tumors of the uterine corpus and gestational trophoblastic disease. In *Atlas of Tumor Pathology*, Rosai J (ed.) pp. 47–83. Armed Forces Institute of Pathology: Washington DC.

WATANABE K, SASANO H, HARADA N, SATO S AND YAJIMA A. (1995). Aromatase in human endometrial carcinoma and hyperplasia. Immunohistochemical, *in situ* hybridization, and biochemical studies. *Am. J. Pathol.*, **146,** 491–500.